# The effect of the type of dietary protein on the development of ovarian cancer

**DOI:** 10.18632/oncotarget.25253

**Published:** 2018-05-08

**Authors:** Ahmed A.A. Taha, Masafumi Koshiyama, Noriomi Matsumura, Kaoru Abiko, Ken Yamaguchi, Jyunzo Hamanishi, Tsukasa Baba, Budiman Kharma, Ibrahim Hassanin Mohamed, Magdy Mohamed Ameen, Salah Ali Ismail, Ikuo Konishi, Masaki Mandai

**Affiliations:** ^1^ Department of Gynecology and Obstetrics, Graduate School of Medicine, Kyoto University, Kyoto, Japan; ^2^ Department of Gynecology and Obstetrics, Sohag Faculty of Medicine Sohag University, Sohag, Egypt; ^3^ Department of Women’s Health, Graduate School of Human Nursing, The University of Shiga Prefecture, Shiga, Japan

**Keywords:** plant protein diet, animal protein diet, cisplatin, IGF-1, p-4EBP1

## Abstract

We evaluated whether different dietary protein qualities (isocaloric diets involving animal (casein) or plant protein (soy protein) could inhibit the ovarian cancer growth in mice and improve their prognosis and whether chemotherapy had different tumor reducing effects on these mice. In the mice of the 20% plant protein group, the ovarian cancer growth at 5 weeks after tumor implantation was clearly reduced in comparison to the mice in the 20% animal protein group (p< 0.001). The serum levels of insulin and IGF-1 levels were both lower in the mice of the 20% plant protein group than in the mice of the 20% animal protein group (p<0.001 and p<0.01, respectively). Immunohistochemistry revealed that the level of eukaryotic initiation factor 4E-binding protein 1 (p-4EBP1) activity―one of the major downstream effectors of the mTOR pathway ―of the plant protein group was significantly weaker than that of the animal protein group (p<0.001). The prognosis of the 20% plant protein group was better than that of the 20% animal protein group (log-rank test, p=0.0062). The ovarian cancer growth in the 20% plant protein plus cisplatin treatment group was not significantly reduced in comparison to the 20% animal protein plus cisplatin treatment group. Our findings suggest that a diet high in plant protein reduces the growth of human ovarian cancer cells in mice compared to a diet high in animal protein, ―possibly through the lack of activation of the IGF/Akt/mTOR pathway, and leads to a better prognosis with or without cisplatin treatment.

## INTRODUCTION

Ovarian cancer is the foremost cause of gynecological cancer-related death in the developed world. Nevertheless, it is the seventh-most commonly diagnosed female cancer worldwide, and second most commonly diagnosed gynecological cancer [1]. It is usually diagnosed at an advanced stage based on the late appearance of symptoms such as abdominal pain, abdominal swelling, gastrointestinal symptoms and pelvic pain [2]. Regardless of the advancements of debulking surgery with additional platinum-based chemotherapy, the outcome remains poor with a 5-year survival rate of <35 % [3].

Lifestyle factors, such as the diet, may influence the pathogenesis of many body cancers [4, 5], including ovarian cancer [6, 7]. However, the role of diet in the development of ovarian cancer is unclear [8], and the active ingredients that are responsible are even less well understood [7]. Various studies have reported positive associations with a low fat intake [9, 10], a high fiber intake [11] and poultry and fish consumption [12] and a low risk of ovarian cancer. A protective effect of fruit and vegetables has also been shown [13]. However, these findings have not been confirmed in other studies [14, 15]. Furthermore, few studies have evaluated the effects of dietary protein sources and amino acid modification upon ovarian tumor growth.

IGF-1, a powerful growth factor, exerts strong effects on each of the key stages of cancer development and higher levels of IGF-1 have been found to be associated with an increased disease risk, tumor metastasis and a poor prognosis in ovarian cancer via the activation of IGF1-R [16]. IGF-1 activates downstream signaling pathways, such as PI3K/Akt/mTOR [17]. Through the stimulation of insulin/IGF-1, the activation of the PI3K/Akt/mTOR pathway results in a profound disturbance of the control of cell growth and survival, which ultimately leads to a competitive growth advantage, metastatic competence, angiogenesis, and resistance to therapy [18, 19].

The major downstream effects of mTOR are the eukaryotic initiation factor 4E-binding protein 1 (4EBP1) and p70S6 kinase (p70S6K) [20, 21]. Activated mTOR increases protein translation through the phosphorylation of 4EBP1 and p70S6K. eIF4E is a eukaryotic translation initiation factor involved in directing ribosomes to the cap structure of mRNAs. It is a 24-kD polypeptide that exists as both a free form and as part of the eIF4F pre-initiation complex [22]. The other subunits of eIF4F are a 47-kD polypeptide known as eIF4A [23] that possesses ATPase and RNA helicase activities and the 220-kD scaffolding polypeptide, eIF4G [24] [25]. The phosphorylation of 4EBP1 by mTOR and various kinases releases eIF4E from 4EBP1, followed by binding of eIF4E, eIF4G and other partners to form eIF4F, which is required for cap-dependent translation [21]. A reduction in the dietary protein intake was reported to be effective for inhibiting tumor growth in prostate and breast cancer, possibly through the inhibition of the IGF/Akt/ mTOR pathway [4] [26].

In the present study, we evaluated whether or not different dietary protein qualities (isocaloric diets involving animal or plant protein) could inhibit the ovarian cancer growth in mice and improve their prognosis. In addition, we evaluated whether or not chemotherapy had different tumor reducing effects on these mice. We then performed an immunoassay to assess whether or not these dietary manipulations could modulate the insulin/IGF production in ovarian cancer. Finally, we investigated whether these dietary manipulations could activate or inhibit the PI3K/Akt/mTOR pathway based on the immunohistochemical expression of p-4EBP1 and the indices of cellular proliferation.

## RESULTS

### The dietary protein quality affected the ovarian cancer growth and progression in the subcutaneous ovarian cancer model

To investigate whether or not dietary protein restriction affected the growth and progression ovarian tumors, we first acclimatized 4- to 6- week-old B6C3F1 mice to either a high (20%) or low (10%) plant or animal protein diet for 2 weeks. Thereafter, we subcutaneously injected HM-1 cells into both sides of the backs of mice. We then measured the body weight weekly and the tumor size twice weekly (preimplantation study).

**Figure 1:**
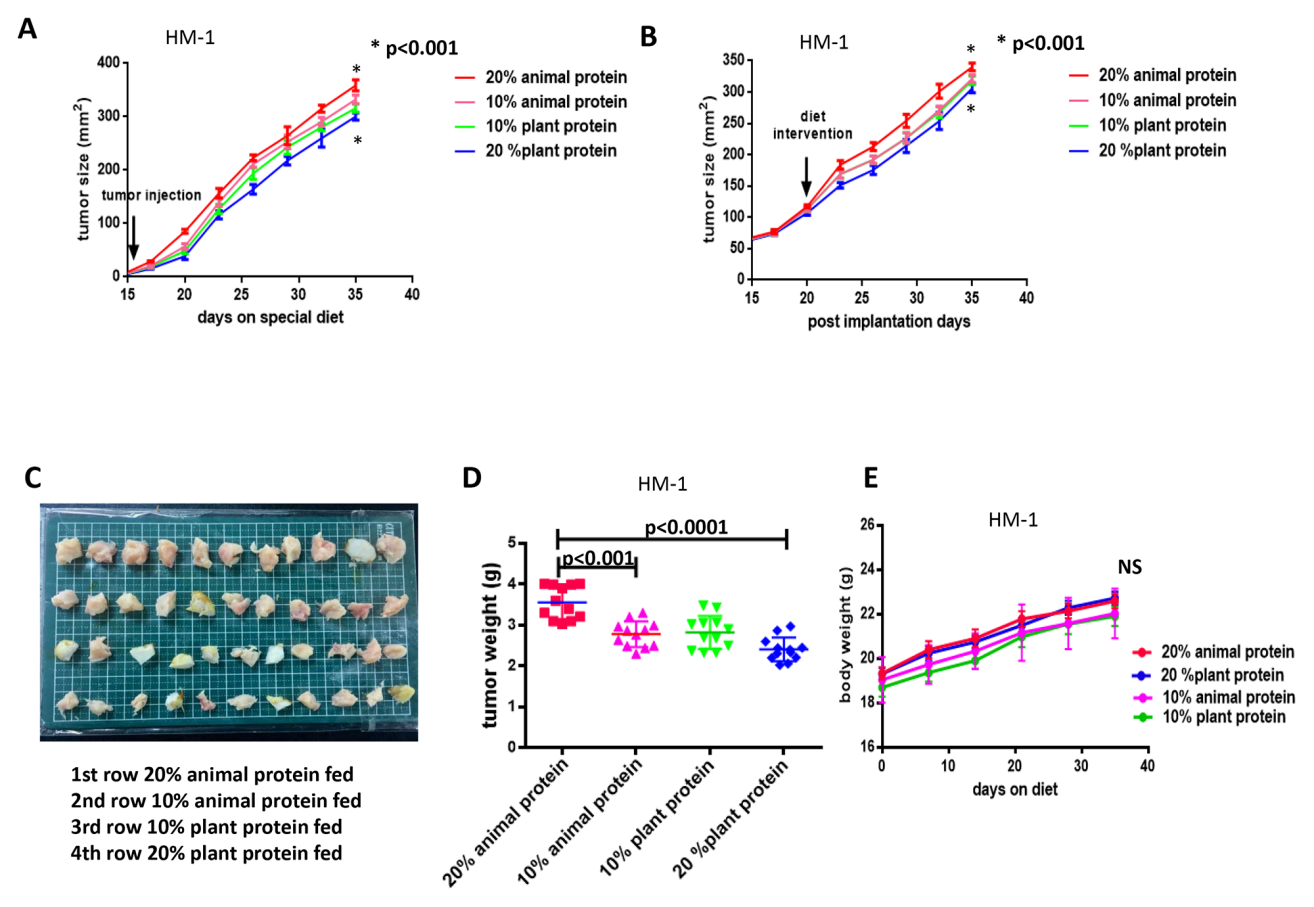
The tumor size, tumor weight and body weight in the 20% and 10% animal protein groups and the 20% and 10% plant protein groups **(A)** The growth of the HM-1 ovarian cancer cells in the 20% plant protein group was significantly lower than that in the 20% animal protein group (p<0.001). **(B)** When protein restriction was initiated after the tumors had reached a size of 100 mm^2^, the results were the same (p<0.001). **(C)** The macroscopic appearance of the resected tumors in the four groups. From the top, the tumors are the 20% animal protein group, 10% animal protein group, 10% plant protein group and 20% plant protein group. **(D)** The average tumor (HM-1) weight in the 20% plant protein was lower than that in the 20% animal protein group (p<0.0001). **(E)** At two months after the start of the experiment, the body weights of the mice in the four groups did not differ to a statistically significant extent.

As shown in Figure 1A, in the mice of the 20% plant protein group, the ovarian cancer growth at 5 weeks after tumor implantation was clearly reduced in comparison to the mice in the 20% animal protein group (p< 0.001). Furthermore, the subcutaneous tumor growth in the 10% animal protein and 20% plant protein groups was decreased in comparison to the 20% animal protein and 10% plant protein groups, respectively (p<0.01). The same results were observed when protein restriction was initiated in the 20% plant protein group or the 20% animal protein group after the tumor reached 100 mm^2^ in size (p<0.001) (post-implantation study; Figure 1B, Figure 1C).

At the end of the experiment, the average tumor weight in the mice of the 20% plant protein group was consistently lower than in the 20% animal protein group (p<0.0001) (Figure 1C, Figure 1D). However, the mean body weights of the groups did not differ to a statistically significant extent over the two-month study period (Figure 1E).

### The dietary protein quality affected the intra-abdominal dissemination of ovarian cancer cells and the survival of the syngeneic ovarian cancer model

To validate our previous results with regard to the effects of dietary protein manipulation on the progression of ovarian cancer, we generated an intraperitoneal ovarian cancer model. We also used ID-8 cells to confirm that the route of peritoneal dissemination of the ID-8 cells and the experimental results were the same as those of the HM-1 cells.

**Figure 2:**
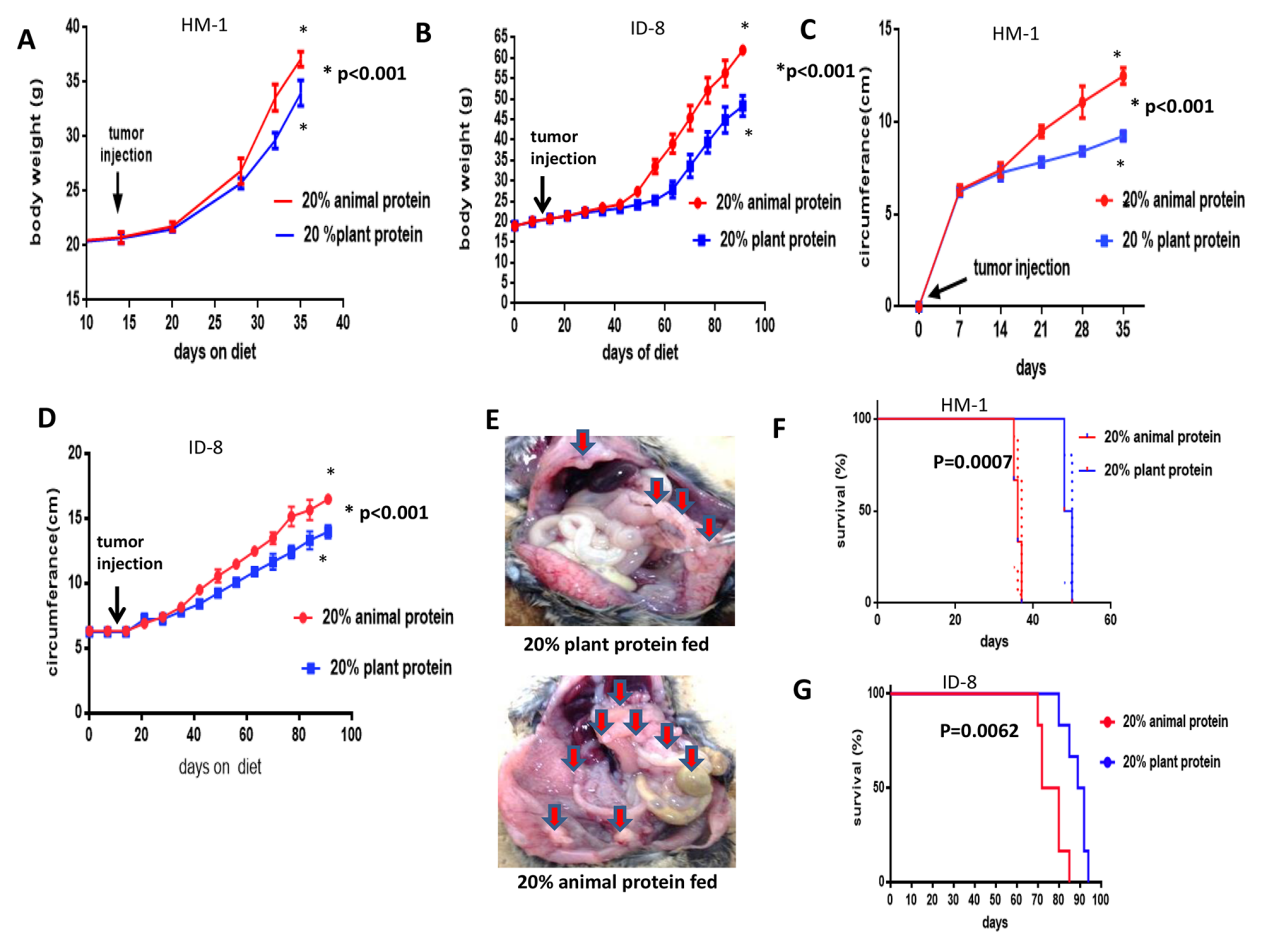
The body weight, abdominal circumference and prognosis of the 20% animal protein and 20% plant protein intraperitoneally injected with HM-1 and ID-8 cells **(A)** Following the injection of HM-1 cells, the body weight of the mice in the 20% animal protein group was significantly higher than in the 20% plant protein group (p<0.001). **(B)** The same results were obtained when the mice were injected with ID-8 cells (p<0.001). **(C)** Following the injection of HM-1 cells, the mice in the 20% animal protein group showed a greater abdominal circumference than the mice in the 20% plant protein group (p<0.001). **(D)** The same result was obtained in mice injected with ID-8 cells (p<0.001). **(E)** The macroscopic appearances of intra-abdominal dissemination in the two groups. The disseminated tumors have a greater volume in the 20% animal protein group than in the 20% plant protein group (arrows). **(F)** Following the injection of HM-1 cells, the prognosis of the mice in the 20% plant protein group was better than that of the mice in the 20% animal protein group (p=0.0007). **(G)** Furthermore, following the injection of ID-8 cells, the prognosis of the mice in the 20% plant protein group was better than that of the mice in the 20% animal protein group (p=0.0062).

The HM-1 and ID-8 cell lines were tested separately by intraperitoneal injection into B6C3F1 and C57BL/6 mice after 2 weeks of special dietary protein feeding (preimplantation study), respectively. The body weights were then measured as an indicator of the tumor and ascites burden in the 2 groups at opposite ends (20% animal protein group vs 20% plant protein group) (Figure 2A, 2B). Similarly to the previous experiments, the mice in the 20% animal protein group had similar average body weights to the mice in the 20% plant protein group until the day of the tumor injection (day 14). After the tumor injection, however, their progressive weight gain (indicative of the tumor burden) changed. At the end of the study, the mice in the 20% animal protein group still had a higher average body weight, while the mice in the 20% plant protein group had a lower average body weight in both studies (both p< 0.001). Similarly, the 20% animal protein group had a larger abdominal circumference than the 20% plant protein group, indicative of the tumor and ascites burden in both studies (all p<0.001) (Figure 2C, 2D). The disseminated tumors have a greater volume in the 20% animal protein group than in the 20% plant protein group (arrows) (Figure 2E).

We also studied the effect of the dietary protein quality on the survival of mice with metastasis from ovarian cancer. Statistically significant differences in the survival were observed between the two dietary protein intervention groups with both the HM-1 and ID-8 cell lines (Figure 2F, 2G). When the HM-1 cell line was used, the median overall survival in the 20% plant protein group was 49 days, while that in the 20% animal protein group was 36 days (log-rank test, p=0.0007) (Figure 2F) When the ID-8 cell line was used, the prognosis of the 20% plant protein group was better than that of the 20% animal protein group (log-rank test, p=0.0062) (Figure 2G).

### The effects of plant or animal protein on the different regulation of insulin or IGF-1 in mice with ovarian cancer

**Figure 3:**
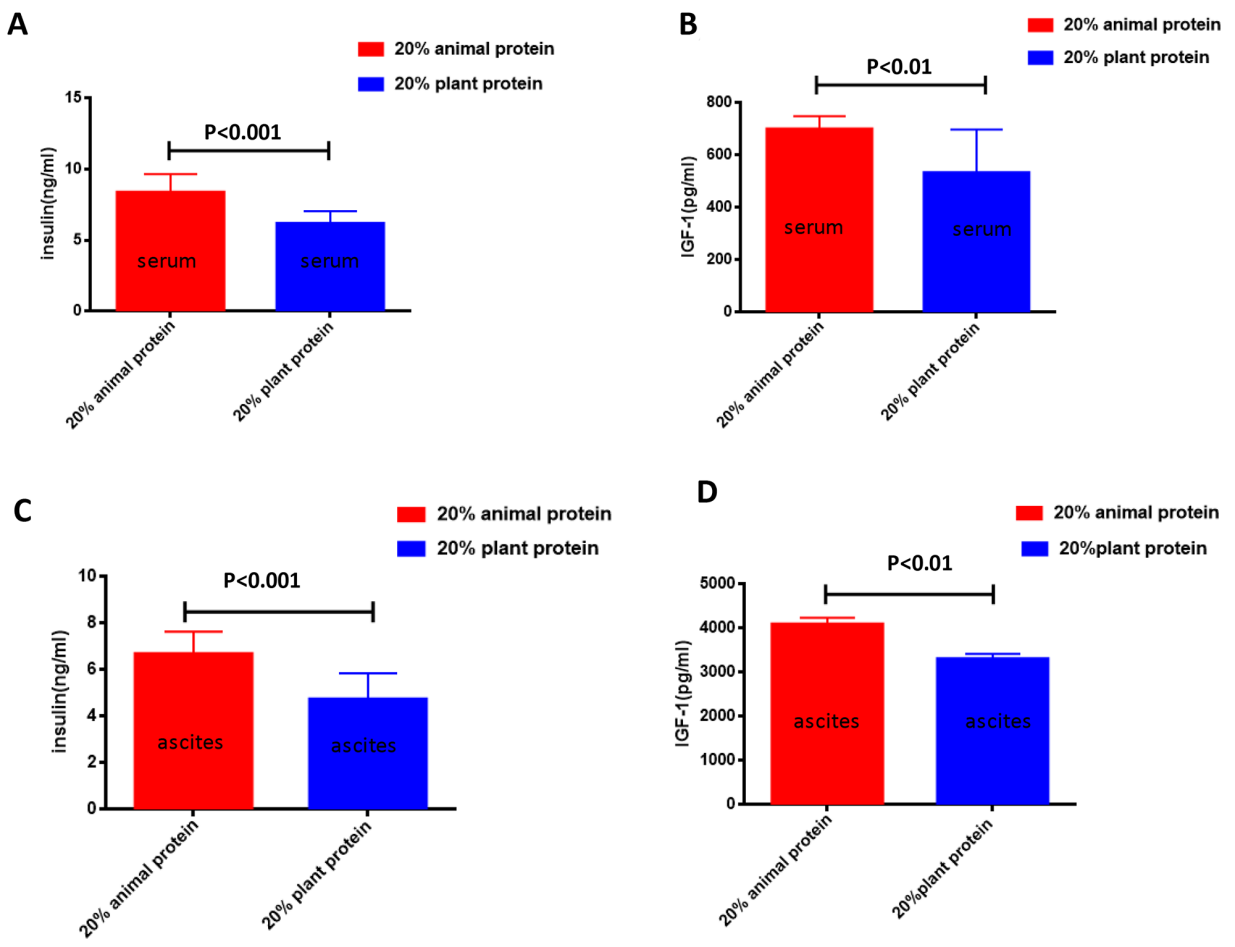
The serum/ascites levels of insulin and IGF-1 in the 20% animal protein group and the 20% plant protein group **(A)** The serum insulin level of the mice in the 20% animal protein group was higher than that of the mice in the 20% plant protein group (p<0.001). **(B)** The same result was observed in the serum level of IGF-1 (p<0.01). **(C)** The level of insulin in the ascites of the 20% animal protein group was higher than that of the 20% plant protein group (p<0.001). **(D)** The same result was observed in the level of IGF-1 in the ascites (p<0.01).

We also analyzed the serum and ascites levels of insulin and IGF-1 in mice with ovarian cancer. The insulin and IGF-1 levels were measured in the serum and ascites of the 20% animal protein and the 20% plant protein groups. The serum levels of insulin and IGF-1 were both lower in the mice of the 20% plant protein group than in the mice of the 20% animal protein group (p<0.001 and p<0.01, respectively) (Figure 3A, 3B). Similarly, the ascites levels of insulin and IGF-1 were both lower in the 20% plant protein group than in the 20% animal protein group (p<0.001 and p<0.01, respectively) (Figure 3C, 3D).

### The effect of animal and plant protein diet on the regulation of the AKT/ mTOR pathway activity in mice with ovarian cancer

**Figure 4:**
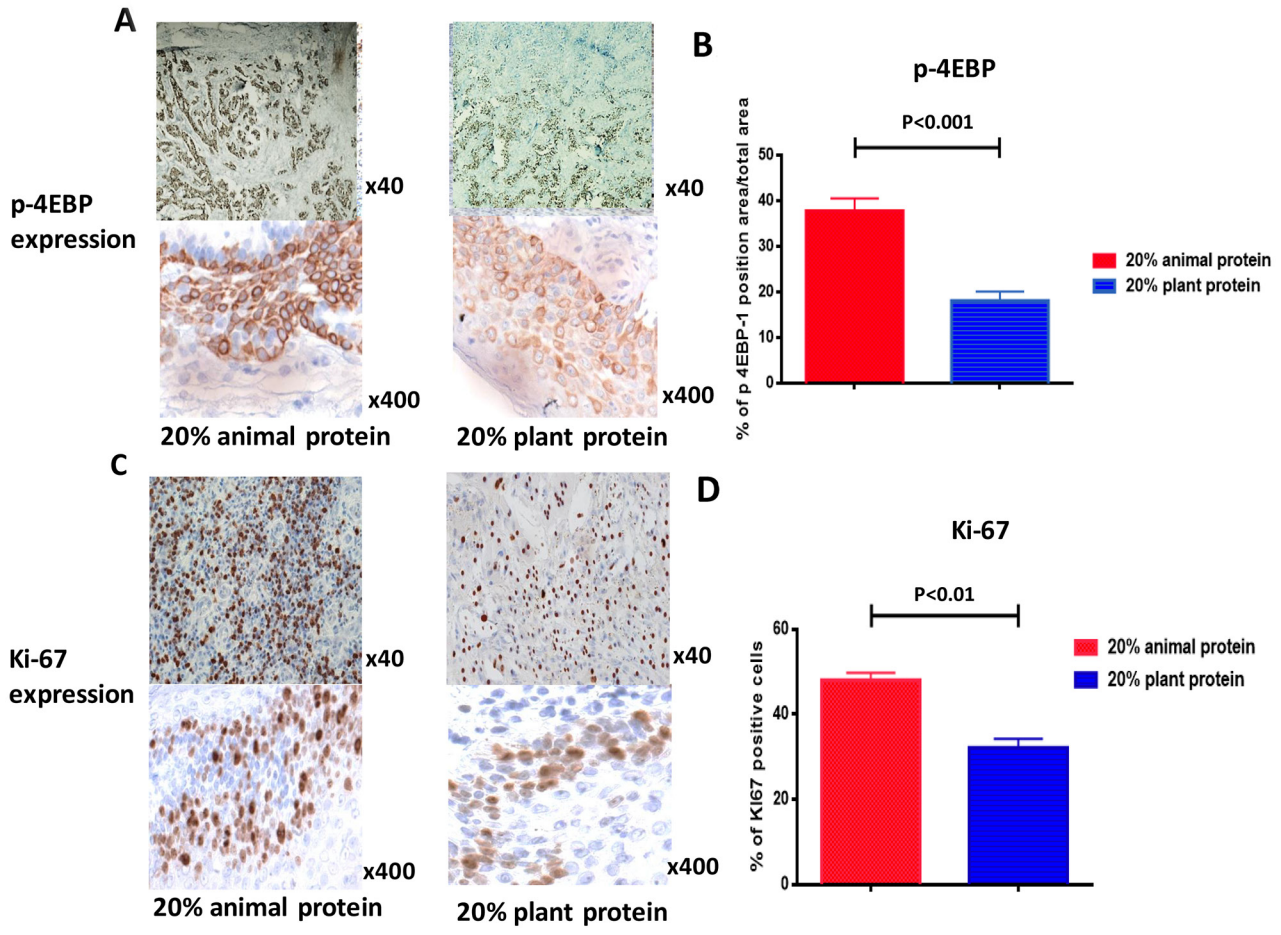
Immuno-histochemical staining for p-4EBP1 and Ki67 in tumors **(A, B)** The rate of p-4EBP1 positivity in the tumors of the 20% animal protein group was higher than that of the 20% plant protein group (p<0.001). **(C, D)** The rate of positivity for Ki67, an index of proliferation, in the tumors of the 20% animal protein group was also higher than that of the 20% plant protein group (p<0.01).

Immunohistochemistry revealed that the level of eukaryotic initiation factor 4E-binding protein 1 (p-4EBP1) activity―one of the major downstream effectors of the mTOR pathway ― of the plant protein group was significantly lower than that of the animal protein group (p<0.001) (Figure 4A, 4B). Immunostaining for Ki-67 showed that the proliferation index of the tumors in the 20% plant protein group was significantly lower than in the tumors of the 20% animal protein group (P<0.01) (Figure 4C, 4D).

### The impact of the type of dietary protein on the efficacy of cisplatin in a syngeneic ovarian cancer model

**Figure 5:**
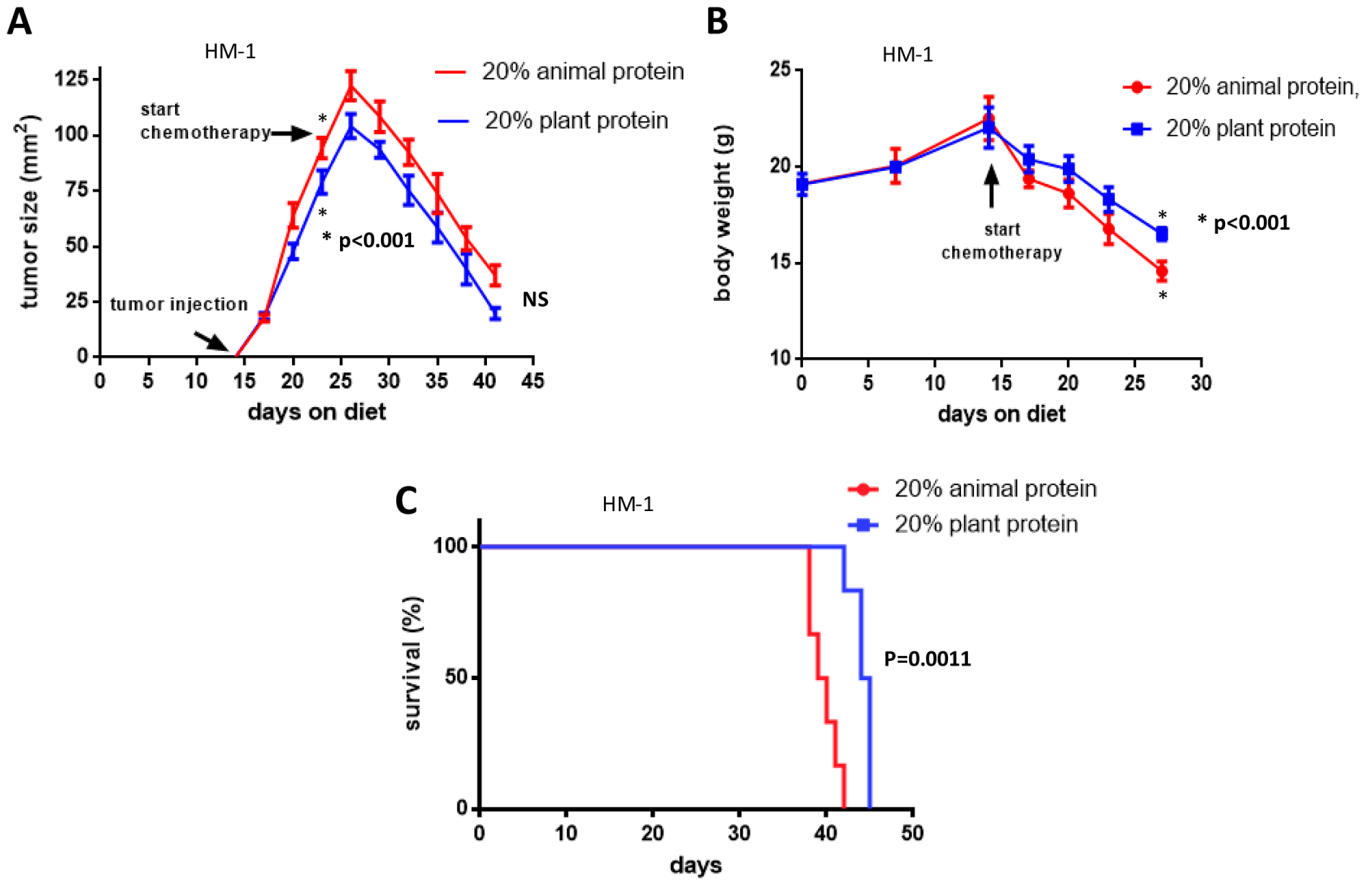
The effects of cisplatin and the types of diet on tumor growth, body weight and the prognosis **(A)** The ovarian tumor growth in the 20% plant protein plus cisplatin treatment group was likely reduced in comparison to the 20% animal protein plus cisplatin treatment group. However, two graphs of the tumor size showed parallel reductions after cisplatin treatment (NS). **(B)** After cisplatin treatment, the 20% plant protein group showed less weight loss than the 20% animal protein group (P<0.001). **(C)** After cisplatin treatment, the 20% plant protein group had a better prognosis than the 20% animal protein group (p=0.0011).

We explored the effects of the types of dietary protein quality with and without cisplatin treatment on the progression of HM-1 ovarian tumor and the response to treatment. As shown in Figure 5A, the ovarian tumor growth in the 20% plant protein plus cisplatin treatment group was likely reduced in comparison to the 20% animal protein plus cisplatin treatment group. However, the rates of tumor reduction after cisplatin treatment were not significantly different between the two groups (NS). Two graphs of the tumor size showed parallel reductions after cisplatin treatment. Interestingly, the plant protein group showed less weight loss after the injection of cisplatin than the animal protein group (p<0.001) (Figure 5B). Furthermore, after treatment with cisplatin, the survival of the mice in the plant protein group was significantly prolonged in comparison to that of the mice in the animal protein group (log-rank test; p=0.0011) (Figure 5C). The median overall survival in the 20% plant protein group was 44.5 days; while that in the 20% animal protein group was 39.5 days.

## DISCUSSION

In the present study, we evaluated the effects of the type and quantity of dietary protein and their influence on the effects of anticancer drug treatment in an animal model of ovarian cancer. Our studies showed that the growth of ovarian cancer in the mice fed plant protein was inhibited in comparison to mice fed animal protein. In addition, the plant protein-fed groups showed a better prognosis than the animal protein-fed groups. In brief, the different types of dietary protein had different effects on the growth of ovarian cancer in mice.

In previous human clinical studies, certain foods have been reported to be associated with a high risk of ovarian cancer, including red meat [27, 28], processed meat [28, 29], saturated fatty acids and animal-based fat [30]. In contrast, the consumption of vegetables [31], particularly green and orange-yellow vegetables [32], has been reported to improve the ovarian cancer survival and certain other types of foods have been reported to reduce the risk of ovarian cancer, including foods that are low in fat [9, 10], foods that are high in fiber [11], poultry [33, 34] and fish [28, 29, 35]. Very recently, a randomized trial of the lifestyle intervention of the Ovarian Cancer Enhanced Survival NRG 0225 study, which promotes the consumption of a high-fiber, low-fat diet that is rich in vegetables along with daily physical activity goals has started. Its hypothesis is that the intervention will improve the progression-free survival (PFS) after oncological therapy for stage II-IV ovarian cancer [36]. The results of our experiments in mice are consistent with these clinical data. However, the mechanisms underlying the different effects of these foods with regard to the growth and prognosis of ovarian cancer remain unclear.

Regarding the representative proteins in the present study, casein and lactalbumin were extracted from milk as animal protein, and soy protein, wheat gluten and corn gluten were extracted as plant protein (Table 1). We initially intended to use casein for most of the animal protein in this study. Plasma levels of IGF-1 are known to be increased by casein intake in humans [37]. Compared with meat-based protein intake, the increased intake of milk-based protein through cow’s milk consumption (200 to 600ml) resulted in a 30% increase in the IGF-1 serum concentration [38]. Therefore, it is reasonable to differentiate between signaling proteins like milk proteins and structural proteins like meat and fish protein, which are less efficient in elevating the insulin and IGF-1 plasma concentrations than milk proteins. IGF-1 is an important activator of PI3K/Akt signaling resulting in mTORC1 activation [39-41].

**Table 1:** The composition and ingredients of 10% or 20% animal protein, and 10% or 20% plant protein diet

Diet composition	20% animal protein	10% animal pro+tein	20% plant protein	10% plant protein
**Total energy value (kcal/g)**	3.7	3.7	3.7	3.7
**Carbohydrate (%Kcal)**	62.93	73.23	63.15	73.06
**Fat (%kcal)**	16.97	16.97	16.87	16.74
**Protein (%Kcal)**	20.11	10.04	19.98	10.20
**Leucine (g/kg)**	18.7	9.4	21.2	10.6
**Isoleucine (g/kg)**	10.1	5.0	7.9	4.0
**Lysine (g/kg)**	15.7	7.9	3.9	1.9
**Methionine (g/kg)**	4.9	2.4	4.1	2.1
**Cysteine (g/kg)**	2.0	1.0	3.0	1.5
**Arginine (g/kg)**	6.7	3.3	7.8	3.9
**Phenylalanine (g/kg)**	8.8	4.4	10.4	5.2
**Tyrosine (g/kg)**	9.2	4.6	5.0	2.5
**Histidine (g/kg)**	4.9	2.4	4.2	2.1
**Threonine (g/kg)**	8.4	4.2	5.5	2.7
**Tryptophan (g/kg)**	2.5	1.2	1.6	0.8
**Valine (g/kg)**	11.8	5.9	8.7	4.3
**Wheat gluten (g/kg)**	―	―	110	55
**Corn gluten (60%) (g/kg)**	―	―	136	68
**Isolated soy protein (g/kg)**	―	―	22	11
**Casein (g/kg)**	170	85	―	―
**Lactalbumin (g/kg)**	44	22	―	―
**Corn starch (g/kg)**	380.1	486.3	325.4	459
**Maltodextrin (g/kg)** **Sucrose (g/kg)**	100 150	100 150	100 150	100 150
**Corn oil (g/kg)**	32	32	32	32
**Olive oil (g/kg)**	32	32	32	32
**Cellulose (g/kg)**	50	50	50	50
**Mineral Mix, w/o Ca & P**	13.14	13.14	13.14	13.14
**Calcium phosphate (g/kg)**	8	10.8	12	12.6
**Calcium carbonate (g/kg)**	10.3	8.3	7.0	6.8
**Vitamin Mix, Tekland (g/kg)**	10	10	10	10

Therefore, compared with the intake of meat, the intake of milk (casein) and whey protein comprising milk products results in a higher and more rapid increase in leucine and insulin signals, which are both important downstream activators of mTORC1. Circulating pulsatile increases in the leucine and insulin concentrations along with permanently elevated serum concentrations of IGF-1 may be responsible for cow milk’s optimized mode of hyperactivated mTORC1 signaling, which amplifies the already upregulated oncogenic mTOR signaling in prostate cancer cells with preexisting alterations of cell growth-promoting signaling pathways [42]. Using prostate and breast cancer cells, Fontana et al. showed that an isocaloric reduction of the animal protein intake significantly reduced the serum IGF-1 concentrations, and inhibited mTOR activity, ―as indicated by the down-regulation of phosphorylated mTOR and p70-S6K [4].

Immunohistochemical analyses of PI3K/AKT/mTOR have shown that this pathway is activated in roughly half of all cases of high-grade serous adenocarcinoma (SAC) of the ovary [43]. Mabuchi et al. examined tissue microarrays of 98 primary ovarian cancers (52 clear cell carcinomas (CCCs) and 46 SACs) and showed that AKT, mTORC1 and mTORC2 are more frequently activated in CCC than in SAC [44]. Therefore, the PI3K/AKT/mTOR pathway may have been more strongly activated by milk (casein) intake than plant protein (soy protein) intake in our mice with ovarian cancer.

Once activated, mTORC1 phosphorylates the translation-regulating factors ribosomal S6 kinase-1 (S6K-1) and eukaryote translation initiation factor 4E binding protein-1 (4EBP-1). The activation of S6K-1 leads to the translation of mRNA encoding ribosomal proteins, elongation factors and other proteins required for transition from the G1 phase to the S phase of the cell cycle. The phosphorylation of 4EBP-1 also enhances the translation of mRNA encoding cyclin D1, c-Myc and hypoxia-inducible factor-1α leading to cell cycle progression or angiogenesis [45]. No et al. reported that the overexpression of p-4EBP1 was associated with a poor prognosis in humans [21]. The ovarian cancer cells in the present study showed higher proliferative activities and reactivity of 4EBP-1 expression in the animal protein groups than in the plant protein groups. In other words, the ovarian cancer cells in mice fed a diet high in plant protein showed lower proliferative activities than those in mice fed a diet high in animal protein.

Next, the consumption of α-lactalbumin (including in milk) as a dietary protein has been reported to have a beneficial effect in controlling breast cancer aggressiveness in comparison to casein fed animals [46]. α-Lactalbumin was also reported to exhibit inherent antiproliferative potential in multiple cancer cells [47]. However, the dose of lactalbumin was about one quarter of that of casein in our experimental food. Therefore, the proliferative potential of casein intake might be much higher than the antiproliferative potential of lactalbumin.

Concerning the activities of wheat gluten, corn gluten and soy protein in our study, we were unable to find any references discussing the relationship between these glutens and cancer development. However, some reports have found that soy protein intake inhibited the proliferative activities of certain cancers. Rayaprolu et al. showed that soybean peptide fractions inhibited *in vitro* human blood (CCRF-CEM and Kasumi-3), breast (MCF-7) and prostate (PC-3) cancer cell lines by up to 68% [48]. Soy milk digestion extract (daizein, glycitein, genistein) might inhibit the proliferation of *in vitro* human prostate cancer cells by regulating the expression of ERβ, PSA, p21, Cyclin D1 and CDK4 in an estrogen receptor (ER)-dependent manner [49]. The soy-derived peptide Vglycin decreased the tumor volume by 38% in xenograft mice transplanted with colon cancer (CT-26) cells [50]. The mechanisms of these phenomena may be due to the down regulation of CDK2 and Cyclin D1, G1/S phase cell cycle arrest and the dysregulated expression of Bax, Bcl-2, and Mcl-1. Mouse xenograft studies have also shown that the soybean-derived peptide lunasin inhibited non-small cell lung cancer cell (NSCLC H1299) volume by 63% at day 32 [51]. This agent inhibited cell cycle progression at the G1/S phase interface by suppressing the phosphorylation of the retinoblastoma protein (RB).

Kolahdooz et al. hypothesized that the fat content of meat, and the fact that meat may contain carcinogenic heterocyclic amines may be associated with the risk of ovarian cancer [12]. They also hypothesized that the high intake of processed meat could increase the risk of ovarian cancer, which is limited via the endogenous formation of N-nitroso compounds. These factors were thought to be associated with the risk of the onset of ovarian cancer. Based on our data, however, we hypothesize that a diet that is high in casein might increase the growth of ovarian cancer cells are naturally present in mice through the activation of the IGF/Akt/mTOR pathway.

Figure 5 compares the cisplatin sensitivities, cachexia and prognosis of the mice after the injection of cisplatin between the animal and plant protein groups. The cisplatin sensitivities were not significantly different between the animal and plant protein groups. Parallel reductions in the tumor size were observed in both groups. However, the plant protein group showed less weight loss after the injection of cisplatin than the animal protein group and a better prognosis. Because the tumors in the plant protein group showed less growth before the injection of cisplatin than the animal protein group, they might not induce loss of the physical strength through all processes. In contrast, the animal protein group is believed to have developed cachexia with weight loss after the injection of cisplatin. Ultimately, the 20% plant protein plus cisplatin treatment group had a better prognosis than the 20% animal protein plus cisplatin treatment group.

Our data also support the hypothesis that the high intake of animal proteins in Western diets might play a role in the development of ovarian cancer, whereas a Japanese traditional diet that is rich in soybean proteins might inhibit the growth of ovarian cancer. In Japan, the age-standardized ovarian cancer mortality rate is only 6.2 per 100,000 women [52]. In contrast, the age-standardized mortality rates in women in the USA and UK were 7.5 and 12.6 per 100,000 women, respectively [53] [54].

In summary, a diet that was high in plant protein reduced the growth of human HM-1 and ID-8 ovarian cancer cells in mice in comparison to a diet that was high in animal protein, ―possibly through the relative inhibition of the IGF/Akt/mTOR pathway. The ovarian cancer cells in the high plant- protein group did not show a greater cisplatin sensitivity than those in the high animal-protein group. However, the animal protein group is believed to have developed cachexia with weight loss after the injection of cisplatin. The prognosis of mice with ovarian cancer fed a diet high in plant protein was better than that of mice with ovarian cancer fed a diet high in animal protein, regardless of cisplatin treatment. In the future, it will be a necessary to invesgate the cell proliferation, cycle and apoptosis and perform other tests *in vitro* in order to elucidate the mechanisms by which plant protein inhibits the progression of ovarian cancer.

## MATERIALS AND METHODS

### Cell lines and culture

The OV2944-HM-1 (derived from B6C3F1 mouse, lymphnode-metastatic ovarian tumor, Riken, Saitama, Japan) and ID8 (derived from C57BL/6 mouse, received from Kansas University, KA, USA) mouse ovarian cancer cell lines were used and cultured in our experiments. The culture medium for HM-1 consisted of minimum essential medium (MEM) alpha (Life Technology, Gibco™, MA) supplemented with 10% heat-inactivated fetal bovine serum (v/v; Biowest, Nuaillé, France) and penicillin–streptomycin (100 IU/ml penicillin, 100 mg/ml streptomycin; Nacalai Tesque, Kyoto, Japan). ID8 cells were maintained in Rosewell Park Memorial Institute (RPMI) media containing 10% (v/v) FBS, which was purchased from (Nacalai Tesque, Kyoto, Japan)

### Animal studies

Female B6C3F1 and C57BL/6 traditional hybrid mice (immunocompetent mice; 4-6 weeks old) were purchased from CLEA Japan Co. (Tokyo, Japan) and maintained under specific pathogen-free conditions. B6C3F1 mice were used for HM1 cell’s experiments, while C57BL/6 mice were used for the ID8 cell experiments. All of the animal experiments were approved by the Kyoto University Animal Research Committee.

### Experimental diet protocols

The four experimental diets were prepared and sterilized via irradiation by Japan CLEA (Tokyo, Japan). The composition and ingredients of each diet are summarized in Table 1. Animal protein included casein and lactalbumin, and plant protein included wheat gluten, corn gluten and soy protein. The mice were allowed free access to food in the cage and an autoclaved water supply via the auto-watering system. The mice were randomized to receive a diet containing 10% or 20% animal protein, or 10% or 20% plant protein.

### Syngenic models of human ovarian cancer

After two weeks of feeding each special diet, two mouse models were generated via the injection of ovarian cancer cell lines into B6C3F1 or C57BL/6 mice. The first was a subcutaneous model, in which HM-1 cells (1 million) were injected into both sides of the backs of B6C3F1 mice; the second was an intraperitoneal model, in which cells from the HM-1 (1 million cells) and ID-8 (5 million cells) cell lines in 200 μl PBS were injected into the peritoneal cavity of B6C3F1 and C57BL/6 mice, respectively. We also used ID-8 cells to confirm that the route of peritoneal dissemination of the ID-8 cells and the experimental results were the same as those of the HM-1 cells.

### The assessment of tumor growth

After the subcutaneous implantation of cancer cells (HM-1), the tumor sizes (n=12 per group) and body weights (n=6 per group) were recorded twice and once a week, respectively. The tumor weight (n=12 per group) was measured using a weighing scale at the end of the experiments. The tumor sizes were assessed by caliper measurements of the two diameters of the tumor (longest length **×** shortest length [mm^2^]).

In the intraperitoneal implantation model (using HM-1 and ID-8 cells; n=6 per group)), the body weight and abdominal circumference were recorded once a week. The total tumor volume, including the number and weight of tumor nodules was identified in various organs. Furthermore, the variation in the volume of the collected ascites was assessed.

### Chemotherapy

To assess the relationship between diet and the synergistic effect of cisplatin inhibitor, mice (n=12 per group) were further randomized within each group to receive either cisplatin (3mg/kg/day for 3 days × 2 weeks) intraperitoneal (IP) chemotherapy or vehicle after the tumor size reached 100 mm^2^. This was a subcutaneous model, in which HM-1 cells (1 million) were injected into both sides of the backs of B6C3F1 mice. After the start of treatment, the tumor size was measured twice weekly, while the body weight of the mice was measured weekly.

### The collection of blood and ascites for the measurement of IGF-1 and the collection of tumors for the histological analyses

At the end of the experiment, blood was collected from the cardiac cavity. The serum was separated, and aliquots were either used to assess the serum glucose level or stored for further analyses at - 80 °C.

The serum insulin and IGF-1 concentrations were measured in duplicate using mouse-specific insulin (Mercodia Mouse Insulin ELISA, Article no 10-1247-10) and IGF-1 (Mouse/Rat IGF-1 Immunoassay Quantikine ELISA Kit, MG100; R&D Systems Inc., MN).

Blood and ascites were collected from the intraperitoneal model to estimate the levels of insulin and IGF-1 by an ELISA. The tumor tissues were excised, weighed and either stored at -80°C or fixed in 10% buffered formalin for the histopathological analysis.

### Immunohistochemistry

Paraffin-embedded tissue sections (4μm) were deparaffinized and rehydrated through a graded series of ethanol washes. Antigen unmasking was achieved by boiling slides under high pressure in a pressure cooker filled with sodium citrate buffer (pH=6.0) for phosphorylated 4EBP1, or Tris–EDTA buffer for (pH=9.0) Ki-67. The sections were further incubated in 3% hydrogen peroxide to reduce endogenous activity. To examine the expression of Ki67 and 4EBP1, tissue sections were blocked and incubated overnight in primary antibodies against Ki67 (1:100; Bethyl Laboratories, Inc., TX), and rabbit monoclonal antibody against phosphorylated 4EBP1 (Thr37/46) (1:100). After primary incubation, the tissue sections were incubated in horseradish-conjugated anti-rabbit antibody according to the manufacturer’s protocol. They were then subjected to enzymatic development in diaminobenzidine (DAB) and counterstained in hematoxylin. The number of positive cells was determined in a blinded fashion by analyzing 4 random 20× fields per tissue and quantified using the Keyence software program (Tokyo, Japan).

### Statistical analyses

The results are presented as the mean ± standard error. The results of the quantitative analyses were plotted on graphs and compared among the groups (animal protein versus plant protein, with or without treatment) using a two-tailed Student’s *t*-test. The serum insulin and IGF-1 concentration and the results of the immunohistochemical analyses were also compared using Student’s *t*-test. The survival curves were compared using the log-rank test. The Graph Pad Prism software program (v.6.02, CA) was used for the statistical analyses. P values of <0.05 were considered to indicate statistical significance.
